# Rationale for sequential extracorporeal therapy (SET) in sepsis

**DOI:** 10.1186/s13054-023-04310-2

**Published:** 2023-02-07

**Authors:** Claudio Ronco, Lakhmir Chawla, Faeq Husain-Syed, John A. Kellum

**Affiliations:** 1 International Renal Research Institute of Vicenza, IRRIV Foundation, Department of Nephrology, Dialysis and Transplantation, St. Bortolo Hospital, aULSS8 Berica, Via Rodolfi, 37, 36100 Vicenza, Italy; 2 Department of Medicine (DIMED), University of Padua, Via Giustiniani, 2, 35128 Padua, Italy; 3 Department of Medicine, Veterans Affairs Medical Center, 3350 La Jolla Village Dr, San Diego, CA 92161 USA; 4 Department of Internal Medicine II, University Hospital Giessen and Marburg, Justus-Liebig-University Giessen, Klinikstrasse 33, 35392 Giessen, Germany; 5 Division of Nephrology, University of Virginia School of Medicine, 1300 Jefferson Park Avenue, Charlottesville, VA 22908 USA; 6 Center for Critical Care Nephrology, CRISMA, Department of Critical Care Medicine, University of Pittsburgh School of Medicine, 3550 Terrace Street, Pittsburgh, PA 15261 USA; 7 Spectral Medical, 135 The West Mall, Unit 2, Toronto, M9C 1C2 Canada

**Keywords:** Adsorption, Cytokines, Endotoxin, Extracorporeal therapies, Sepsis, Septic shock, Viral infection

## Abstract

**Abstract:**

Sepsis and septic shock remain drivers for morbidity and mortality in critical illness. The clinical picture of patients presenting with these syndromes evolves rapidly and may be characterised by: (a) microbial host invasion, (b) establishment of an infection focus, (c) opsonisation of bacterial products (e.g. lipopolysaccharide), (d) recognition of pathogens resulting in an immune response, (e) cellular and humoral effects of circulating pathogen and pathogen products, (f) immunodysregulation and endocrine effects of cytokines, (g) endothelial and organ damage, and (h) organ crosstalk and multiple organ dysfunction. Each step may be a potential target for a specific therapeutic approach. At various stages, extracorporeal therapies may target circulating molecules for removal. In sequence, we could consider: (a) pathogen removal from the circulation with affinity binders and cartridges (specific), (b) circulating endotoxin removal by haemoperfusion with polymyxin B adsorbers (specific), (c) cytokine removal by haemoperfusion with sorbent cartridges or adsorbing membranes (non-specific), (d) extracorporeal organ support with different techniques for respiratory and cardiac support (CO_2_ removal or extracorporeal membrane oxygenation), and renal support (haemofiltration, haemodialysis, or ultrafiltration). The sequence of events and the use of different techniques at different points for specific targets will likely require trials with endpoints other than mortality. Instead, the primary objectives should be to achieve the desired action by using extracorporeal therapy at a specific point.

**Graphical Abstract:**

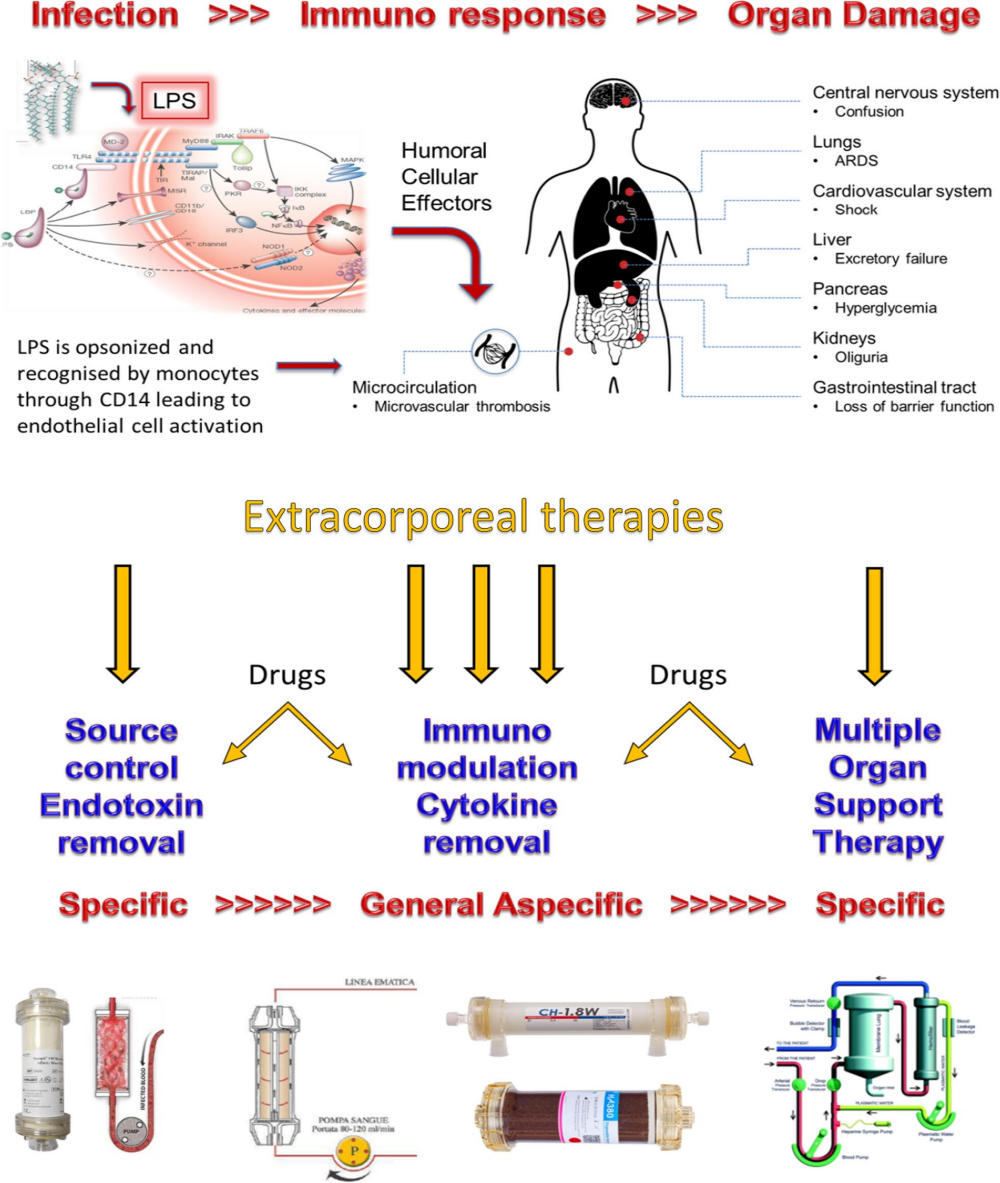

## Introduction

**Fig. 1:**
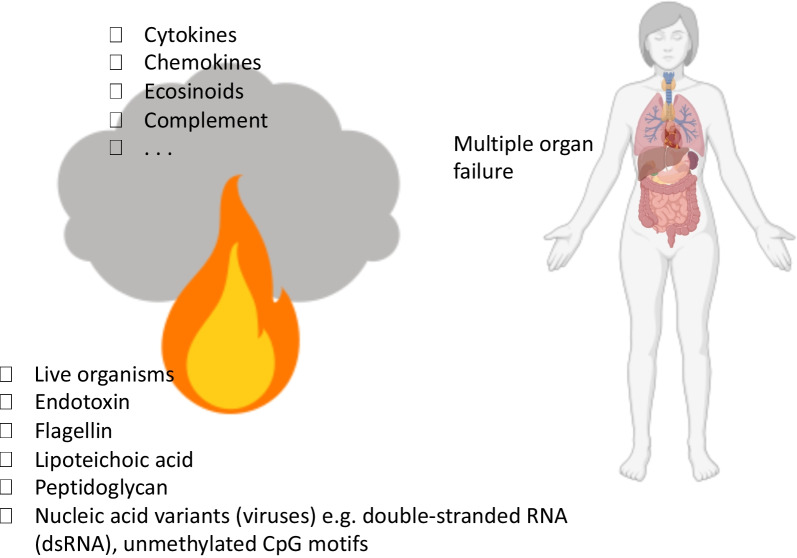
Triggers and mediators of organ failure in sepsis. The figure illustrates some triggers of the systemic inflammatory and immune responses in sepsis. An insult (e.g. uncontrolled infection, circulatory shock, tissue necrosis, apoptosis) causes the release of a variety of mediators, which can be microbial and host glycoproteins, lipoproteins, and nucleic acids. Sepsis is then initiated upon host recognition of the mediators, when an overwhelming systemic proinflammatory response is generated and the compensatory anti-inflammatory responses fail to rebalance the systems to homeostasis. The effects of this inappropriate response to the trigger may lead to cellular dysfunction and ultimately organ failure. Notably, the risk factors for the development of sepsis and organ dysfunction likely include comorbidities and host genetic factors in addition to pathogen-related factors

Before the advent of antibiotics, infections were often treated surgically with a focus on source control. Previously, drainage and amputation were often life-saving interventions for treating septicaemia. For the past 7 decades, the approach to sepsis treatment has been on source control and has focused on the timely administration of effective antibiotics and vasoactive agent management, among others [1, 2]. This approach is largely treatment by ‘addition’: the administration of a medicine. The antibiotic-first mindset has been hugely successful; nonetheless, many patients die from sepsis/septic shock despite standard source control and broad-spectrum antibiotics. For these patients, the presence of the pathogen itself, pathogen products (e.g. bacterial DNA and endotoxin), and high plasma levels of cytokines directly contribute to poor outcomes (Fig. 1) [3–5].

Extracorporeal treatment for sepsis/septic shock is fundamentally an intervention of subtraction. Blood is removed from the body and through a filter and/or haemoadsorber, where the pathogen, pathogen-related products, and/or cytokines are removed [6]. In essence, subtractive therapy removes substances the body cannot effectively clear, which accelerates repair and recovery. Sepsis is defined as life-threatening organ dysfunction caused by a dysregulated host response to infection [7]. Accordingly, sepsis features three critical components: (1) host invasion by the pathogen, (2) the host response, and (3) organ failure [8]. Each component can be targeted by existing therapies; blood purification is uniquely suited to address all three components. For example, whole pathogens or pathogen-associated molecular patterns (PAMPs), such as endotoxin, can be removed from the bloodstream using various haemoperfusion devices [9]. Although this is an initial step in the development of sepsis, the host response follows rapidly and the host is usually completely engaged at presentation [5]. While pathogen and PAMP clearance typically resolve host response, toxic inflammation and other perturbations may persist and represent viable targets [5, 10]. A challenge is that numerous targets exist, and therefore, any strategy for treatment must be broad spectrum. Finally, extracorporeal therapies have a history in organ support, especially for managing kidney, heart/lung, and liver failure [11]. In the present review, we will explore these therapies in a context we term sequential extracorporeal therapy (SET).

## Pathogenesis of sepsis

**Fig. 2:**
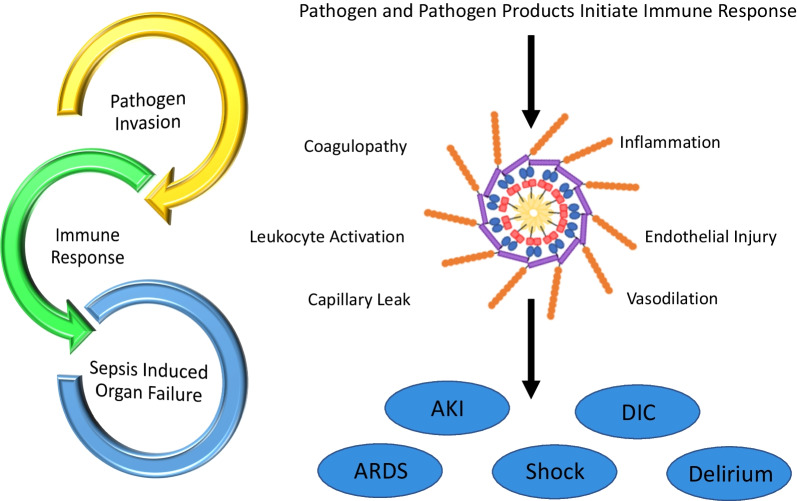
Immune response following pathogen invasion. Pathogen invasion and pathogen products, such as endotoxins, can trigger the initial sepsis cascade, evoking both innate and cell-mediated immune responses. Numerous factors may contribute to a dysregulated host response to infection, including upregulation of pro-inflammatory and anti-inflammatory cytokines, leukocyte activation, damage-associated molecular patterns released by injured cells, and host-specific factors such as age, comorbidities, and genetic characteristics. Escalation of the dysregulated immune response in the form of coagulopathy and excessive inflammation leads to endothelial injury and capillary leak, oedema formation, and compromised innate defence against invading microorganisms, which eventually leads to multiple organ failure. *AKI* acute kidney injury, *ARDS* acute respiratory distress syndrome, *DIC* disseminated intravascular coagulation

Sepsis involves physiologic, pathologic, and biochemical abnormalities caused by infection [12]. Pathogen and pathogen compound invasion trigger a complex series of biologic responses, and the infection source/type, even if local, often dictates the sequence and timing of the host biologic and immune responses. Once infected, the host innate immunity is activated by the effective recognition of the pathogen, PAMPs, or damage-associated molecular patterns (DAMPs) [8]. Driven largely by pattern-recognition receptors (PRRs) activated by DAMPs and PAMPs, the elements of innate immunity initiate a broad immune response that upregulates inflammatory pathways and mediators designed to orchestrate an effective response to an infectious agent [13]. However, in many patients, the immune response itself causes tissue injury, immune dysregulation, mitochondrial damage, and coagulation disorders that in turn results in a self-sustaining vicious cycle of inflammatory responses potentially leading to organ failure and death (Fig. 2) [14]. A full review of sepsis is beyond the scope of this review. Therefore, we will focus on key mediators that worsen outcomes. Removing these mediators can improve outcomes. Table 1 provides an overview of the currently available extracorporeal blood purification (EBP) cartridges.

**Table 1:** Selection of currently available extracorporeal blood purification devices

Device name (manufacturer)	Sorbent type	Intended use	Toxins/mediators removed
Seraph 100 Microbind Affinity Blood Filter (ExThera Medical)^a^	Polyethylene beads with end-point-attached heparin	Septic shock	Bloodstream pathogens, drugs
Toraymyxin (Estor; Toray)^a^	Polymyxin B covalently bound to polypropylene-polystyrene fibre	Septic shock	Endotoxin
Cytosorb (Cytosorbents)^a^	Crosslinked Divinylbenzene/polyvinylpyrrolidone copolymers	Septic shock, vasoplegic shock (e.g. post-cardiac surgery, extracorporeal membrane oxygenation), liver failure, rhabdomyolysis, intoxication, drug accumulation	Cytokines, myoglobin, free haemoglobin, bilirubin/bile acids, toxins, metals, drugs
HA330/380 (Jafron Biomedical)^b^	Polystyrene divinylbenzene copolymer resins	Sepsis, trauma, burns, liver failure, rhabdomyolysis, intoxication, drug accumulation	Cytokines, myoglobin, free haemoglobin, bilirubin/bile acids, toxins, metals, drugs
oXiris (Baxter)^c^	AN69 with PEI surface treatment; endotoxin adsorbed by means of ionic interactions at the membrane surface	AKI, sepsis	Uremic toxins, endotoxin, cytokines
SepXiris (Baxter)^d^	AN69-ST copolymer membrane	AKI, sepsis	Uremic toxins, cytokines
Haemofeel CH (Toray)^e^	PMMA membrane	AKI, sepsis	Uremic toxins, cytokines

*AKI* acute kidney injury, *AN69* acrylonitrile and sodium methylal sulfonate copolymer membrane, *PEI* polyethyleneimine, *PMMA* polymethyl methacrylate, *ST* surface treated

^a^Available in Europe and Japan, and only for compassionate or emergency use in USA

^b^Available in China and Europe

^c^Available in China and Europe, and only for compassionate or emergency use in USA

^d^Available in Europe and Japan

^e^Available in Europe and Japan

### Pathogenemia

Local infection is one of the sources of systemic dissemination of pathogens, pathogen compounds, and products of damaged tissue into the bloodstream. Pathogenemia refers to an infective agent measurable in the blood. For patients with sepsis, pathogenemia is associated with worse outcomes [15, 16]. Furthermore, the pathogen load is directly linked to higher morbidity and mortality [17–19]. In the antibiotic era, antimicrobial administration was taken for granted. However, with evolving resistance and the emergence of new pathogens without effective therapies, clinicians are encountering patients with persistent pathogenemia more frequently.

#### Extracorporeal removal of pathogens

The rationale for pathogen removal is straightforward. Pathogens cause host injury and the pathogen itself is an effective stimulus of PRRs [20]. Before 2018, reducing bloodstream pathogen was largely for patients with malaria and babesiosis. In these blood-centred infections, whole blood exchange is performed to debulk the infectious load [21]. The Seraph-100 haemoadsorber uses heparin as a surface to bind pathogens. Heparin/heparan sulphate proteoglycans are used as essential factors in binding and virulence in many pathogenic infections (Table 2) [22, 23]. As heparin and heparan sulphate are nearly identical surfaces, Seraph-100 has a broad capacity to remove various pathogens effectively [24].

**Table 2:** Heparan sulphate proteoglycan–pathogen interactions in infection

Pathogen	Pathogen protein	Heparan sulphate proteoglycan	Function/interaction
*Bacteria*			
Chlamydia pneumoniae	OmcB	Unknown	Attachment
Haemophilus influenzae	High molecular weight protein	Unknown	Attachment
Listeria monocytogenes	ActA	Syndecan-1	Attachment, invasion
Pseudomonas aeruginosa	LasA	Syndecan-1	Shedding
*Staphylococcus aureus*	α-toxin, ß-toxin	Syndecan-1	Shedding
*Streptococcus pyogenes*	M protein	Unknown	Attachment
*Streptococcus pneumoniae*	ZmpC	Syndecan-1	Shedding
*Viruses*			
Adenovirus	Ad3 Fibre knob	Unknown	Attachment
Coronavirus	Spike protein	Unknown	Attachment
Cytomegalovirus	gB	Unknown	Attachment
Dengue virus	Envelope protein	Unknown	Attachment, internalisation
Herpes simplex virus-1 and -2	gB, gC, gD	Syndecan-2	Attachment
*Parasites*			
Plasmodium spp.	Circumsporozoite protein	Multiple	*Plasmodium* circumsporozoite protein cleavage, productive invasion
Trypanosoma cruzi	Cruzipain	Heparan sulphate proteoglycan	Enhanced enzymatic activity
	Heparin-binding protein	Unknown	Attachment

Adapted from Barlett et al. [21]. The table summarises some of the many bacterial, viral, and parasitic infections that subvert cell surface heparan sulphate proteoglycans during infection

Animal studies on Seraph-100 sponsored by the US Defense Advanced Research Programs Agency have demonstrated its safety, broad-spectrum pathogen elimination ability, and clinical efficacy [24]. In pre-clinical studies, Seraph-100 treatment has been demonstrated to be useful for the removal of drug-resistant and susceptible gram-positive bacteria [25], carbapenem-resistant gram-negative bacteria [26], and viruses [24, 27]. Depending on the organism, between 30% (*Klebsiella pneumoniae*) and 99.9% (carbapenem-resistant *K. pneumoniae*) of the targeted pathogen can be removed per pass of blood over Seraph-100 heparinised media [24]. For virus removal, between 50% (adenovirus) and 90% (cytomegalovirus) of the targeted virus can be removed per pass [24]. Furthermore, a recent case series on seven critically ill patients with COVID-19 reported an ~ 10% reduction in the level of SARS-CoV-2 nucleocapsid protein after treatment with the Seraph-100 device [28].

In addition to pathogens, several DAMPs also bind to Seraph-100 heparinised media. These include histones, nucleosomes, heparin-binding protein, lipopolysaccharide (LPS)-binding protein, high mobility group box 1 (HMGB1), and platelet factor 4 (PF4) [29, 30].

A severe pneumonia nonhuman primate study [31] reported that Seraph-100-treated animals demonstrated significantly reduced *Streptococcus pneumoniae* PAMPs, which reduced kidney injury, metabolic acidosis, and hypoglycaemic shock. Furthermore, renal oxidative injury and NLRP3 inflammasome activation were reduced. Further, bronchoalveolar lavage CCL2 and 4, and interleukin (IL)-18 were attenuated compared to the controls.

A case study reported effective clearance of persistent *S. aureus* infection by Seraph-100 with a single 4-h treatment, followed by negative blood cultures, despite several days of empirical antibiotic therapy previously that had not been effective [32]. A recent in vitro circulation model showed that the technology reduces aminoglycoside plasma levels by approximately 60% [33]. Clinical case reports have shown that the Seraph-100 does not affect remdesivir [34] or vancomycin, tacrolimus, and mycophenolic acid concentrations [35].

More than 800 patients have been treated with Seraph-100 primarily for COVID-19 in Europe and the USA. In COVID-19, RNAaemia appears to be a risk factor for COVID-19 disease deterioration and severity [36–39]. A study detected SARS-CoV-2 viremia in 100% intensive care unit (ICU) patients [37] and determined that a plasma RNA level > 6000 copies/mL was strongly associated with mortality. In another study, Seraph-100 significantly reduced the circulating concentration of SARS-CoV-2 nucleocapsid protein in COVID-19 patients, suggesting virus removal from the blood [28]. The US Department of Defense funded an observational clinical trial to evaluate Seraph-100 efficacy for treating COVID-19 (ClinicalTrials.gov, NCT04606498). Using preliminary data from the first 99 patients (53 treated patients and 46 controls), a nearly fourfold improved survivability odds was observed for Seraph-100-treated patients versus controls [40]. Data from a COVID-19 registry documenting Seraph-100-treated patients also demonstrated improved survival [41]. The most significant finding was that survival was associated with treatment within 60 h of ICU admission, during which the SARS-CoV-2 concentration was the highest in the blood [41].

### PAMPs

Several molecules derived from pathogenic organisms can trigger inflammation and activate the complement system. Endotoxin (i.e. LPS) is the archetypical PAMP. Endotoxin is a stabilising molecule in the outer membrane of the gram-negative bacterial cell wall, which is highly immunostimulatory in humans than in other mammals [42]. This is perhaps surprising as the human intestine transports enormous quantities of endotoxin—more than a million times (i.e. 10–50 g) the lethal dose if administered intravenously [43]. Gram-negative infections may result in endotoxaemia, which antibiotics may release as they kill bacteria [44]. In addition, sepsis leads to a compromised barrier integrity of the gastrointestinal tract, which promotes bacterial products crossing the dysfunctional barrier and eventually resulting in endotoxaemia [45].

Endotoxin can trigger all the cardinal features of sepsis and is likely a modulating factor during sepsis. Endotoxin is recognised by multiple cell types. Endotoxin-induced immune cell activation produces inflammatory proteins and produces direct cytotoxic effects potentially resulting in organ failure. Tissue damage not only from pathogens, but also from the action of immune effector cells can release various DAMPs, which in turn propagate inflammation, often through the same receptors (e.g. Toll-like receptors [TLR]) that recognise PAMPs [46]. This process ensures that even if the endotoxin exposure is transient, the immune response will be robust and long-lasting.

#### Extracorporeal removal of PAMPs

While nonspecific blood purification can eliminate some PAMPs, various specific technologies are under development. The best-known and well-studied of these techniques is polymyxin haemoperfusion that involves endotoxin removal. Polymyxins are a group of cyclic cationic polypeptide antibiotics with well-characterised endotoxin-binding properties [47]. Although toxicity limits the clinical use of polymyxin B as an antibiotic, polymyxin B can bind to a haemoperfusion column, and the circulating endotoxin is effectively removed through exposure to immobilised polymyxin B without systemic toxicity [47]. This method has been available in Japan since 1994 and received CE marking in 1998. More than 100,000 patients have been treated in multiple countries [48]. Clinical data from a national Japanese database analysed using propensity matching demonstrated benefit in the range of 3–7% absolute risk reduction for hospital mortality [49, 50]. No clinical trials have been adequately powered to determine an effect size in this range. The two largest trials to date, i.e. ABDOMIX [51] and EUPHRATES [52], did not report a survival benefit with polymyxin B haemoperfusion. However, only the EUPHRATES trial used the endotoxin activity assay (EAA) to identify appropriate patients for the treatment.

EAA, an FDA-approved and CE-marked immunoassay, uses anti-lipid A monoclonal antibody and whole blood. Endotoxin in the blood binds with the antibody, and the antigen–antibody complex stimulates neutrophils in the sample. Neutrophil-induced reactive oxygen species are then measured by a luminol chemiluminescence reaction. Basal and maximally stimulated samples are measured in parallel as negative and positive controls, respectively, and endotoxin activity in the sample is expressed as a relative value (EAA level) [53]. An EAA level ≥ 0.60 is considered the threshold for high endotoxin activity and is associated with increased ICU mortality [54]. Enrolment into the EUPHRATES trial [52] was restricted to patients with septic shock who had EAA levels ≥ 0.60.

Overall, even in the per protocol analysis of the EUPHRATES trial that was restricted to patients with a multi-organ dysfunction score > 9, the 28-day mortality was 33% with haemoadsorption versus 36.4% with sham, although the difference was not statistically significant [52]. However, the EAA cannot precisely quantify circulating endotoxin when EAA levels are ≥ 0.90, and such values may not represent treatable levels [55]. A reanalysis of the EUPHRATES trial data revealed that 17% patients had EAA levels ≥ 0.90. After excluding these patients, the 28-day mortality was 26.1% for polymyxin B haemoperfusion versus 36.8% for sham (risk difference 10.7%, odds ratio 0.52 (95% CI 0.27–0.99), *P* = 0.047) [56]. These findings prompted the design of an ongoing US trial (ClinicalTrials.gov, NCT03901807).

### DAMPs and mediators

DAMPs have physiologic intracellular roles, but develop additional functions in the extracellular space [57]. DAMPs alert the host to danger and stimulate an inflammatory response, also known as danger-associated molecular patterns or alarmins [46]. In addition to passive release by dead/dying cells [57, 58], some DAMPs are secreted in response to stress [57]. DAMP-induced inflammation is a fundamental component of the immune response that mitigates the pathological effects of injury/infection. However, the loss of balance between inflammation and counter-inflammation caused by the immunodysregulation in sepsis may cause DAMP overproduction, and consequently, excessive mediators may spill-over into the systemic circulation resulting in adverse effects. DAMPs may be released from various intracellular and extracellular components such as the cytosol, nucleus (HMGB1, IL-1a, histones), cytoplasmic vesicles (RNA), extracellular matrix (heparan sulphate, fibronectin), and membranes (Table 3) [59, 60]. As DAMPs provide such a robust inflammatory stimulus, their removal may also represent an important strategy for improving outcomes in sepsis.

**Table 3:** Source and release mechanisms of DAMPs

	Origin	Release mechanism	PRRs
*Intracellular*			
ATP	Mitochondria	Injury	P2X_7_
heat shock proteins	Cytosol	Apoptosis, necrosis	TLR2, TLR4, CD91
Histones	Nucleus	Apoptosis, necrosis	TLR2, TLR4, NLRP3
HMGB1	Nucleus, autophagosome	Apoptosis, necrosis, injury	TLR2, TLR4, TLR9, RAGE
mtDNA	Mitochondria	Trauma, injury	TLR9
*Extracellular*			
Biglycan	Proteoglycan	MMP cleavage, de novo synthesis	TLR2, TLR4, NLRP3
Decorin	Proteoglycan	MMP cleavage, de novo synthesis	TLR2, TLR4
Fibrinogen	Extracellular matrix glycoprotein	Extravasation	TLR4
Fibronectin	Extracellular matrix glycoprotein	Metalloprotease, splicing, unfolding	TLR2, TLR4
Heparane sulphate	Glycosaminoglycan	Heparanase cleavage	TLR4, RAGE

Adapted from Frevert et al. [59]. The table summarises a selection of DAMPs that promote both adaptive and innate immunity signalling and inflammation. DAMPs represent endogenous sterile stimuli, which are released either from dying cells (e.g. histones, HMGB1) or the extracellular matrix (e.g. fibrinogen, fibronectin) and act through direct interaction with TLRs. The activation of TLRs triggers inflammation through the production of proinflammatory mediators and the recruitment of leukocytes to infection and injury sites

*CD* cluster of differentiation, *DAMPs* damage-associated molecular patterns, *HMGB1* high-mobility group protein B1, *mtDNA* mitochondrial DNA, *NLRP3* NOD-like receptor pyrin domain-containing 3, *PRRs* pattern recognition receptors, *RAGE* receptor for advanced glycation end products, *TLRs* toll-like receptors

HMGB1 and adenosine triphosphate (ATP) represent the archetype of DAMPs. HMGB1 is a chromatin protein involved in DNA chaperoning [57] and is expressed in almost all cell types; and HMGB1 converts to a DAMP when transferred into the extracellular space. Interestingly, HMBG1 is passively released during necrosis but not apoptosis [61] and is also secreted during severe stress [57]. Once in the extracellular environment, HMGB1 via paracrine signalling activates both innate and adaptive immunity through multiple receptors (e.g. advanced glycation end products, TLR4) [62]. ATP is a DAMP largely through its activation of purinergic P2 receptors [63] that have widespread expression and are involved in both adaptive and innate immunity. The P2 receptor subset P2YR has been linked with chronic inflammation [57]. Apoptotic cells release ATP, which acts as a chemotropic factor that binds P2YR on macrophages, stimulating their phagocytic activity [64].

Histones are intranuclear cationic proteins present in all eukaryotic cells and are highly conserved across species. Within the nucleus, histones provide structural stability to chromatin and regulate gene expression. Histones may be released into the extracellular space as free, DNA-bound nucleosome, or part of neutrophil extracellular traps (NETs). All three forms are detected in the serum after significant cellular death such as sepsis, trauma, ischemia–reperfusion injury, and autoimmune disease [65, 66]. Once in the extracellular space, histones act as DAMPs, activating the immune system and causing endothelial and epithelial cytotoxicity by interacting with TLRs, complement, and cell membrane phospholipids [57, 67]. Although NETs contribute to pathogen clearance, excessive NET formation promotes inflammation and tissue damage in sepsis [59].

In summary, damaged tissues release a large array of DAMPs, which propagate both adaptive and innate immunity signalling and inflammation. Reducing DAMPs may modulate the inflammatory response and mitigate the effects of a dysregulated response that worsens outcomes.

#### Extracorporeal removal of DAMPs

HMGB1, histones, and histone-decorated NETs are positively charged and therefore, bind to heparin. Recent in vitro data support the notion that haemoadsorption devices (e.g. Seraph-100) can remove these substances effectively. Ebeyer-Masotta et al. [68] demonstrated that heparin-functionalised adsorbents efficiently depleted activated platelets, platelet-derived extracellular vesicles, PF4, HMGB1, and histones/nucleosomes. Similarly, Hogwood et al. [69] demonstrated that heparin attenuates histones and NETs-induced inflammatory responses in whole blood. Furthermore, Wen-Sheng et al. [70] showed that the HA330 cartridge has a significant DAMP removal capacity in patients with sepsis. More research is required, but preliminary data suggest that haemoadsorption may effectively decrease the concentrations of clinically important DAMPs.

Once the pathogen and its products have exerted a certain action on the organism, a cascade of effects ensues in the evolution of the sepsis syndrome. In particular, the cascade of events continues with the opsonisation of LPS by a lipoprotein-binding protein and the complex is recognised via specific patterns (e.g. CD14 receptors) [71]. Subsequently, the signal activates intracellular pathways (e.g. NF-κB) and upregulates a specific pattern response by producing chemical mediators typical of innate and adoptive immunity [72]. According to Hotchkiss and Karl [73], the immunoresponse in sepsis is dysregulated, and endothelial damage and critical illness may develop because of overwhelming inflammation. However, in sepsis, late deaths may also occur due to excessive response via adaptive immunity or immunoparalysis [74]. These phenomena are mediated by various chemical species including proinflammatory and anti-inflammatory cytokines. Such molecules (e.g. IL-1, TNF-α, IL-6, and IL-10) represent the key to endothelial damage and generalised (endocrine) effects at the level of distant organs such as the heart, lungs, and kidneys. The consequent organ damage and pathological organ crosstalk may further aggravate the syndrome and lead to increased risk of mortality [14].

Similar to host pathogen invasion and PAMP dissemination, systemic dissemination-induced immunodysregulation of chemical mediators is potentially mitigated by blockade or extracorporeal removal. However, while the first phases may allow for specific interventions, the immunodysregulation phase presents significant challenges owing to the heterogeneity of mediators involved in the immunoresponse [3, 5]. Therefore, previous attempts to block specific mediators failed to demonstrate clinical efficacy [75]. In fact, attempts to approach the problem with a purely anti-inflammatory drug or blockade of one specific cytokine may be inadequate for achieving meaningful results because pathological processes continue via alternative pathways [76]. Here, the nonspecific nature of extracorporeal adsorption may be advantageous. This approach is justified and supported by the peak concentration hypothesis published in 2003 [77]. In fact, haemoperfusion with new sorbents may remove higher quantities of mediators that present the highest concentration in the blood in a specific timeframe of the syndrome. By removing the excess circulating mediators (both proinflammatory and anti-inflammatory), the therapy may restore a certain degree of immune homeostasis and the immunosystem may restore its capacity to balance between the innate and adaptive responses.

#### Haemoadsorption

**Fig. 3:**
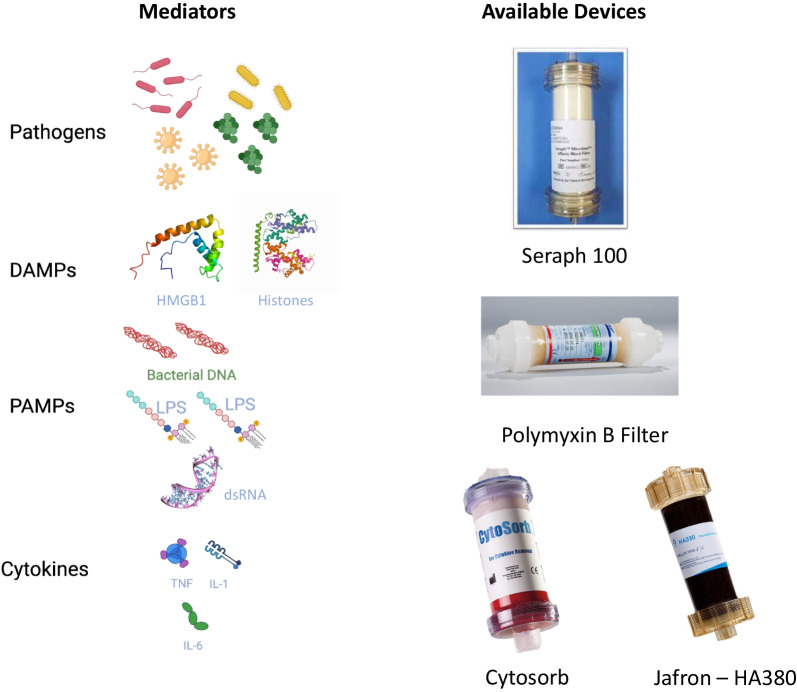
Mediators of sepsis and extracorporeal devices. The figure illustrates a schematic overview of key mediators involved in sepsis pathogenesis and the available extracorporeal blood purification devices targeting the mediators. In the future, enrichment strategies (e.g. genetic signature, molecular biomarkers) may enable a more patient-customised extracorporeal therapy tailored to the underlying biology of sepsis. *PAMPs* pathogen-associated molecular patterns, *DAMPs* damage-associated molecular patterns

The concept that sepsis is associated with the excess mediators that cause organ injury, and that cytokine levels are associated with death risk is well-established and represents the rationale for extracorporeal therapies [5, 78]. Cytokines have molecular weights beyond the cut-off limit of classic dialysis membranes; therefore, sorbent use is indicated. Previously, haemoadsorption presented major adverse effects, rendering its clinical application problematic. New sorbents are more biocompatible, are packed into disposable cartridges easily used in extracorporeal circuits, and are used as a stand-alone device or in conjunction with other extracorporeal therapies (Fig. 3) [79]. Direct haemoadsorption is technically simple and efficient for cytokine removal [80]. An increasing body of evidence has justified further studies on direct haemoadsorption [79, 81]. Sorbents have been used as rescue or adjuvant therapy in sepsis, and experience regarding technique and safety has accumulated. In particular, biochemical effects (significant reduction in circulating cytokines), biological effects (improved HLA-DR expression, restored monocyte function), and clinical effects (improved scale of functioning score, improved haemodynamics, acute kidney injury (AKI) severity mitigation) have been observed [79, 82, 83]. Further studies on technical parameters (e.g. adsorption isotherms for different molecules, including antibiotics, blood flow, anticoagulation, dose prescription, treatment monitoring, marker molecules, and study endpoints) are ongoing (ClinicalTrials.gov, NCT04580680). These studies will help define prescription criteria, the clinical indications for these therapies and their optimal operative characteristics, and the cost–benefit ratio in a detailed health technology assessment process.

In the literature, direct haemoadsorption has been conducted with two types of sorbent units: CytoSorb® (CytoSorbents Corp., Monmouth Junction, NJ, USA) and Jafron HA380 (Jafron Biomedical, Guangdong, China). In a multicentre randomised trial that compared CytoSorb with conventional care, 100 patients with sepsis were randomised to daily CytoSorb haemoperfusion or conventional treatment [84]. Although the CytoSorb device reduced IL-6 levels by 5%–18%, no significant differences in IL-6 levels were observed between the two groups [84], comparable to the results of a recent propensity score-matching study [85]. Furthermore, two randomised trials in patients with infective endocarditis undergoing cardiopulmonary bypass [86] and COVID-19 with vasoplegic shock and multiple organ failure [87] did not demonstrate reductions in vasopressor requirement, organ dysfunction, or mortality. Finally, in a recent single-centre, randomised trial, patients with COVID-19-acute respiratory distress syndrome (ARDS) initiated with veno–venous extracorporeal membrane oxygenation (ECMO) were allocated to CytoSorb haemoperfusion vs. no adsorption [88]. No difference in 72-h IL-6 concentrations was reported between the groups, and haemoadsorption was associated with increased 30- and 90-day mortality. The observed negative findings could be explained by low IL-6 levels at baseline as compared to other studies that reported more efficient IL-6 clearance [89]. Therefore, haemoperfusion might be associated with the consecutive failure to detect a reduction in circulating cytokines. Furthermore, initiating haemoadsorption in a patient already receiving ECMO may ineffective and too late when the primary aim is to reduce cytokine levels and the associated outcomes, respectively.

Haemoperfusion with Jafron HA cartridges (HA330/HA380) has been performed in acute inflammatory conditions (e.g. sepsis, trauma, burns, and pancreatitis). In a recent study involving patients with sepsis, haemoadsorption was associated with improved haemodynamics, reduced IL-8 and IL-6 levels, and reduced ICU length of stay and mortality, compared to the controls [90]. A second randomised trial included 46 patients with ARDS [91]. Haemoperfusion with HA330 significantly decreased TNF-α and IL-1 levels and continuous renal replacement therapy (CRRT) intensity and improved lung injury markers and 28-day mortality [91]. Based on these findings, an ongoing German HA380-sepsis trial has been designed (ClinicalTrials.gov, NCT04306419).

Surface-modified haemodiafilters provide greater adsorptive capacity than conventional polysulfone-based haemodiafilters and, thus, are increasingly being used for the treatment of patients with sepsis and AKI who are on CRRT. The PMMA (polymethyl methacrylate) haemodiafilter is a synthetic polymeric membrane with a symmetric microporous structure that is able to adsorb small- and medium-sized molecules, including cytokines [92–94]. Notably, however, the PMMA membrane has half of the adsorption capacity of the AN69-ST (acrylonitrile and sodium methylal sulfonate copolymer membrane-surface treated) membrane for HMGB-1 [95]. Currently, data on the PMMA haemodiafilter in patients with sepsis is limited to small clinical trials [96, 97].

The heparin-coated oXiris haemodiafilter is an AN69 membrane with a PEI surface coating and has been proposed for removal of cytokines and endotoxins [79, 98]. In a randomised trial involving patients with sepsis-associated AKI, CRRT with oXiris was associated with decreased endotoxin, TNF-alpha, and IL-6 levels as compared to CRRT using a standard high-flux haemodiafilter [100]. Moreover, norepinephrine administration decreased with oXiris but not with the standard filter [100]. In another study on critically ill patients with COVID-19, treatment with oXiris was associated with a reduction in IL-6 levels, improvement in multiorgan function scores, and reduction in expected APACHE IV-derived ICU mortality rate [99]. Notably, the best improvement in mortality rate was observed in patients receiving EBP early on during their ICU stay [99]. In aggregate, the data so far indicate that early treatment in the correct clinical context may be an important factor for maximising treatment effectiveness and potentially preventing progression to multiple organ dysfunction.

## Multiple organ dysfunction and sequential extracorporeal therapy

Sepsis involves a sequence of biological events that lead to organ dysfunction; this sequence presents specific windows of therapeutic opportunity. Accordingly, EBP strategies may be considered in sequence or as separate entities according to the pathophysiological status, as changes in pathophysiological parameters over the disease course might indicate the need for different treatment approaches. With this approach, a multitude of mediators and pathogen/pathogen products can be eliminated to improve outcomes.

While classic membrane-based separation processes, such as haemofiltration and haemodialysis, have significant limitations because of their inability to effectively clear molecules that are more than 20,000 Da in size, new sorbent devices and haemodiafilters with adsorptive properties can be used for specific and nonspecific systemic removal of target molecules, respectively. There is increasing evidence for the benefits of these devices, but more research is required in this field. To start with, the effective capacity of a given sorbent to remove a target molecule needs to be investigated. This should be followed by investigations into the biological effects of target molecule removal, and finally, a series of clinical endpoints should be identified for the therapies. The findings of these studies would provide sufficient information for subsequent studies on improving survival in specific patient populations. However, a single intervention is unlikely to have such a dramatic effect on outcome, and the use of the abovementioned techniques in sequence is recommended. For example, the first phase of pathogen invasion of the host and PAMP dissemination may require specific interventions (i.e. source control by antibiotics and Seraph-100, and endotoxin removal by polymyxin B haemoperfusion), while the immunodysregulation phase may require a broader approach due to the heterogeneity of the mediators involved in the immunoresponse (i.e. removal of DAMPs and mediators by Seraph-100, Cytosorb, and HA330/380) (Graphic Abstract). If a cascade of events occurs, adequate biomarkers and biomonitoring techniques are required to determine the optimal timing for appropriate techniques, in addition to historical knowledge of the syndrome. Finally, in the case of organ failure, organ-specific supportive therapy would be required. For example, patients with severe AKI treated with RRT may benefit from the use of haemodiafilters with adsorptive properties. Haemoadsorption systems can be added, as necessary and in sequence, for the treatment of patients with early-stage sepsis and can be coupled with organ support provided by haemofiltration, ECMO, or other techniques. Based on this idea, measuring key mediators at different points in critically ill patients with sepsis may allow for use of various haemoadsorption techniques independently or in combination with a CRRT or ECMO circuit.

Despite the strong pathophysiological rationale for the use of EBP in sepsis, evidence for its use is limited at present. In contemporary medicine, EBP therapy is based largely on clinical experience and performed in the context of clinical trials, as there is no consensus on the use or the thresholds of specific clinical criteria for initiating, monitoring or discontinuing EBP. Currently, EAA can be used to identify patients who require PMX haemoperfusion, and any positive blood culture or molecular test for the virus can be used to select patients with indications for Seraph-100 therapy. In the future, additional biomarker studies are needed to evaluate the suitability of a patient for bedside EBP therapy, and parameters for monitoring and discontinuing treatments also need to be measured.

Future trials should assess whether combined or sequential EBP techniques can achieve meaningful biological or clinical end points. Based on the findings of the studies summarised above, future randomised controlled trials should first assess different primary endpoints rather than mortality in order to shed light on other important effects of EBP, such as the number of ventilation-free days, vasopressor therapy-free days, and ICU-free days. Further, selecting homogenous patient populations by utilising enrichment strategies (based on genetic signature and molecular biomarkers) is likely to increase the efficacy of EBP trials and the likelihood of obtaining positive results [101]. Further research is also needed to better define and improve the selectivity of target solutes, as increased mediator clearance by haemoadsorption may be accompanied by loss of antibiotics and other medications, which may counter the beneficial effects of EBP techniques and may play an important role in determining patient outcomes. Finally, the high cost of EBP devices needs to be justified in terms of clinical effectiveness (i.e. reduction in hospital-centred outcomes), particularly in times of economic restraints.

## Conclusions

The study of EBP strategies in sepsis reflects progressive understanding of human pathophysiology and host–microorganism interactions. Consequently, new extracorporeal devices have been developed and are readily available in clinical practice. However, despite the strong rationale for extracorporeal strategies, current evidence is insufficient to recommend routine use in all patients meeting requirements. Targeted patient selection for extracorporeal therapies is becoming increasingly clear based on objective measurements (i.e. timing of treatment initiation, patient inclusion criteria), and this approach may more likely translate into improved outcomes if applied in future trials. Furthermore, the sequence of events and use of different techniques at different points for specific targets will likely require sepsis trials with endpoints different from mortality. Rather, the primary objectives should be to achieve the desired action by the extracorporeal therapy used at a specific point.

## Data Availability

Not applicable.

## Abbreviations

AKI: Acute kidney injury
ARDS: Acute respiratory distress syndrome
ATP: Adenosine triphosphate
CD: Cluster of differentiation
COVID-19/SARS-CoV-2: Coronavirus disease 2019/Severe acute respiratory syndrome coronavirus 2
CRRT: Continuous renal replacement therapy
DAMP: Damage-associated molecular pattern
DNA: Deoxyribonucleic acid
EAA: Endotoxin activity assay
EBP: Extracorporeal blood purification
ECMO: Extracorporeal membrane oxygenation
HLA: Human major histocompatibility complex
HMGB1: High mobility group box 1
ICU: Intensive care unit
IL: Interleukin
LPS: Lipopolysaccharide
NET: Neutrophil extracellular trap
NHP: Nonhuman primate
NK-κB: Nuclear factor kappa-light-chain-enhancer of activated B cells
PAMP: Pathogen-associated molecular pattern
PF4: Platelet factor 4
PRR: Pattern-recognition receptor
RNA: Ribonucleic acid
SET: Sequential extracorporeal therapy
TLR: Toll-like receptor
